# (*Z*)-2-(5-Methyl-2-oxoindolin-3-yl­idene)-*N*-phenyl­hydrazinecarbothio­amide

**DOI:** 10.1107/S1600536811049154

**Published:** 2011-11-30

**Authors:** Amna Qasem Ali, Naser Eltaher Eltayeb, Siang Guan Teoh, Abdussalam Salhin, Hoong-Kun Fun

**Affiliations:** a School of Chemical Sciences, Universiti Sains Malaysia, Minden, Penang, Malaysia; b Faculty of Science, Sabha University, Libya; c Department of Chemistry, International University of Africa, Khartoum, Sudan; d X-ray Crystallography Unit, School of Physics, Universiti Sains Malaysia, 11800 USM, Penang, Malaysia

## Abstract

In the title compound, C_16_H_14_N_4_OS, the dihedral angle between the nine-membered 5-methyl­indolin-2-one ring system and the benzene ring is 10.21 (7)°. Intra­molecular cyclic N—H⋯O and C—H⋯S hydrogen-bonding inter­actions [graph set *S*(6)] are present within the N—N—C—N chain between the ring systems. In the crystal, mol­ecules form centrosymmetric cyclic dimers through pairs of N—H⋯O hydrogen bonds [graph set *R*_2_^2^(8)].

## Related literature

For related structures, see: Qasem Ali *et al.* (2011 ▶); Ferrari *et al.* (2002 ▶); Pervez *et al.* (2010 ▶); Ramzan *et al.* (2010 ▶). For the biological activity of Schiff bases, see: Bhandari *et al.* (2008 ▶); Bhardwaj *et al.* (2010 ▶); Pandeya *et al.* (1999 ▶); Sridhar *et al.* (2002 ▶); Suryavanshi & Pai (2006 ▶). For the cytotoxic and anti­cancer activiy of isatin and its derivatives, see: Vine *et al.* (2009 ▶). For bond-length data, see; Allen *et al.* (1987 ▶). For graph-set analysis, see Bernstein *et al.* (1995 ▶).
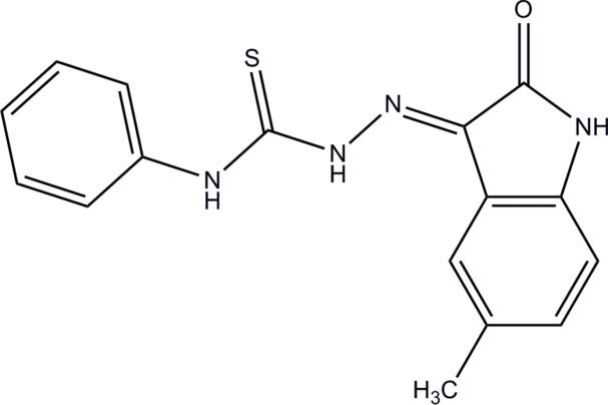


## Experimental

### 

#### Crystal data

C_16_H_14_N_4_OS
*M* *_r_* = 310.37
Monoclinic, 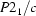

*a* = 5.6875 (3) Å
*b* = 17.9405 (8) Å
*c* = 14.5658 (6) Å
β = 91.105 (3)°
*V* = 1485.97 (12) Å^3^
*Z* = 4
Mo *K*α radiation
μ = 0.23 mm^−1^
*T* = 100 K
0.37 × 0.14 × 0.09 mm

#### Data collection

Bruker APEXII CCD diffractometer
Absorption correction: multi-scan (*SADABS*; Bruker, 2005 ▶) *T*_min_ = 0.921, *T*_max_ = 0.980
25266 measured reflections
4645 independent reflections
3565 reflections with *I* > 2σ(*I*)
*R*_int_ = 0.080

#### Refinement

*R*[*F* ^2^ > 2σ(*F* ^2^)] = 0.054
*wR*(*F* ^2^) = 0.123
*S* = 1.06
4645 reflections
212 parameters
H atoms treated by a mixture of independent and constrained refinement
Δρ_max_ = 0.40 e Å^−3^
Δρ_min_ = −0.30 e Å^−3^

### 

Data collection: *APEX2* (Bruker, 2005 ▶); cell refinement: *SAINT* (Bruker, 2005 ▶); data reduction: *SAINT*; program(s) used to solve structure: *SHELXS97* (Sheldrick, 2008 ▶); program(s) used to refine structure: *SHELXL97* (Sheldrick, 2008 ▶); molecular graphics: *SHELXTL* (Sheldrick, 2008 ▶); software used to prepare material for publication: *SHELXTL* and *PLATON* (Spek, 2009 ▶).

## Supplementary Material

Crystal structure: contains datablock(s) I, global. DOI: 10.1107/S1600536811049154/zs2160sup1.cif

Structure factors: contains datablock(s) I. DOI: 10.1107/S1600536811049154/zs2160Isup2.hkl

Supplementary material file. DOI: 10.1107/S1600536811049154/zs2160Isup3.cml

Additional supplementary materials:  crystallographic information; 3D view; checkCIF report

## Figures and Tables

**Table 1:** Hydrogen-bond geometry (Å, °)

*D*—H⋯*A*	*D*—H	H⋯*A*	*D*⋯*A*	*D*—H⋯*A*
N1—H1*N*1⋯O1^i^	0.89 (2)	1.96 (2)	2.848 (2)	173 (2)
N3—H1*N*3⋯O1	0.91 (2)	2.04 (2)	2.7595 (17)	135.8 (19)
C11—H11*A*⋯S1	0.95	2.63	3.2712 (18)	125

Symmetry code: (i) 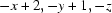.

**Fig. 1.**
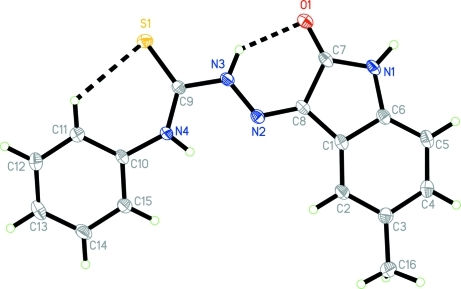
The molecular conformation of the title compound, with 50% probability displacement ellipsoids and the atom-numbering scheme.

**Fig. 2.**
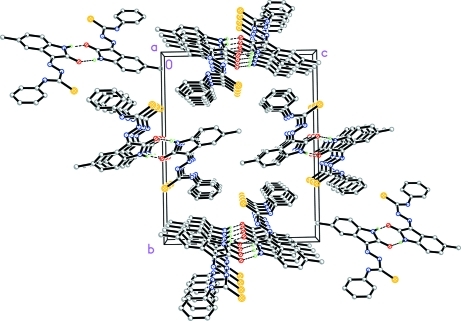
The crystal packing of the title compound viewed down the a axis of the unit cell. Hydrogen bonds are shown as dashed lines.

## supplementary crystallographic information

### Comment

Isatin (2,3-dioxindole) is an endogenous compound identified in humans, and its effect has been studied in a variety of systems. Biological properties of isatin and its derivatives include a range of actions in the brain and offer protection against certain types of infections, such as antibacterial (Suryavanshi & Pai, 2006) antifungal, anticonvulsant, anti-HIV (Pandeya *et al.*, 1999), anti-depressant and anti-inflammatory activities (Bhandari *et al.*, 2008). Recently, we reported the crystal structure of (*Z*)-2-(5-chloro-2- oxoindolin-3-ylidene)-*N*-phenylhydrazinecarbothioamide (Qasem Ali *et al.*, 2011). In the present paper we describe the single-crystal X-ray diffraction study of title compound, C_16_H_14_N_4_OS.

In this compound (Fig. 1), the dihedral angle between the nine-membered 5-methylindolin-2-one ring system and the benzene ring is 10.21 (7)°. The atoms C8 in the 5-methylindolin-2-one ring and C10 in the benzene ring are connected by a chain of four atoms (N2/N3/C9/N4)) giving a torsion angle of 7.3 (2)°, while the torsion angles (C8/N2/N3/C9) and (C10/N4/C9/N3) are 173.20 (15)° and -177.56 (16)°, respectively. These values are very close to those in the previously mentioned analogous structure (Qasem Ali *et al.*, 2011). The essentially planar conformation of the molecule is maintained by cyclic intramolecular N3—H···O1 and C11—H···S1 hydrogen-bonding interactions [graph set *S*(6) (Bernstein *et al.*, 1995)] (Table 1) together with an *S*(5) N4—H···N2 interaction.

In the crystal the molecules form centrosymmetric cyclic dimers through intermolecular N1—H···O1^i^ hydrogen bonds [graph set *R*^2^_2_(8)] (Table 1) (Fig. 2). Weak C—H···π interactions are also present: C5—H5A···*Cg3*^ii^ = 3.6506 (19) Å; C12—H12A···*Cg2*^iii^ = 3.6600 (19) Å. [*Cg3*^ii^ is the centroid of the C10—C15 ring; *Cg2*^iii^ is the centroid of the C1—C6 ring: symmetry code: (ii) = -*x* + 1, *y* + 1/2, -*z* + 1/2; (iii) = -*x*, *y* - 1/2, -*z* + 1/2].

### Experimental

The title compound was synthesized by refluxing the reaction mixture of 4-phenyl-3-thiosemicarbazide (0.01 mol) and 5-methylisatin (0.01 mol) in 60 ml of ethanol for 2 hrs. The precipitate formed during reflux was filtered, washed with cold EtOH and recrystallized from hot EtOH: yield 80%. The orange crystals (m.p. 511.8–512.3 K) were grown in acetone-DMF (3:1) by slow evaporation at room temperature.

### Refinement

H atoms bound to N were located in a difference-Fourier map and were refined freely. The remaining H atoms were positioned geometrically and refined using a riding model with C—H = 0.95 Å (aryl) and 0.98 Å (methyl) and *U*_iso_(H) = 1.2*U*_eq_(aryl C) and *U*_iso_(H) = 1.5*U*_eq_(methyl C). The highest residual electron density peak (0.397 eÅ^-3^)is located at 0.71 Å from C8 and the deepest hole (-0.303 eÅ^-3^) is located at 1.33 Å from C6.

### Crystal data

**Table:**

C_16_H_14_N_4_OS	*F*(000) = 648
*M**_r_* = 310.37	*D*_x_ = 1.387 Mg m^−^^3^
Monoclinic, *P*2_1_/*c*	Melting point = 511.8–512.3 K
Hall symbol: -P 2ybc	Mo *K*α radiation, λ = 0.71073 Å
*a* = 5.6875 (3) Å	Cell parameters from 4470 reflections
*b* = 17.9405 (8) Å	θ = 3.0–30.2°
*c* = 14.5658 (6) Å	µ = 0.23 mm^−^^1^
β = 91.105 (3)°	*T* = 100 K
*V* = 1485.97 (12) Å^3^	Block, orange
*Z* = 4	0.37 × 0.14 × 0.09 mm

### Data collection

**Table:**

Bruker APEXII CCD diffractometer	4645 independent reflections
Radiation source: fine-focus sealed tube	3565 reflections with *I* > 2σ(*I*)
graphite	*R*_int_ = 0.080
φ and ω scans	θ_max_ = 30.9°, θ_min_ = 1.8°
Absorption correction: multi-scan (*SADABS*; Bruker, 2005)	*h* = −8→8
*T*_min_ = 0.921, *T*_max_ = 0.980	*k* = −25→22
25266 measured reflections	*l* = −20→20

### Refinement

**Table:**

Refinement on *F*^2^	Primary atom site location: structure-invariant direct methods
Least-squares matrix: full	Secondary atom site location: difference Fourier map
*R*[*F*^2^ > 2σ(*F*^2^)] = 0.054	Hydrogen site location: inferred from neighbouring sites
*wR*(*F*^2^) = 0.123	H atoms treated by a mixture of independent and constrained refinement
*S* = 1.06	*w* = 1/[σ^2^(*F*_o_^2^) + (0.044*P*)^2^ + 0.9076*P*] where *P* = (*F*_o_^2^ + 2*F*_c_^2^)/3
4645 reflections	(Δ/σ)_max_ < 0.001
212 parameters	Δρ_max_ = 0.40 e Å^−^^3^
0 restraints	Δρ_min_ = −0.30 e Å^−^^3^

### Special details

**Table:**

Geometry. All e.s.d.'s (except the e.s.d. in the dihedral angle between two l.s. planes) are estimated using the full covariance matrix. The cell e.s.d.'s are taken into account individually in the estimation of e.s.d.'s in distances, angles and torsion angles; correlations between e.s.d.'s in cell parameters are only used when they are defined by crystal symmetry. An approximate (isotropic) treatment of cell e.s.d.'s is used for estimating e.s.d.'s involving l.s. planes.
Refinement. Refinement of *F*^2^ against ALL reflections. The weighted *R*-factor *wR* and goodness of fit *S* are based on *F*^2^, conventional *R*-factors *R* are based on *F*, with *F* set to zero for negative *F*^2^. The threshold expression of *F*^2^ > σ(*F*^2^) is used only for calculating *R*-factors(gt) *etc*. and is not relevant to the choice of reflections for refinement. *R*-factors based on *F*^2^ are statistically about twice as large as those based on *F*, and *R*- factors based on ALL data will be even larger.

### Fractional atomic coordinates and isotropic or equivalent isotropic displacement parameters (Å^2^)

**Table:**

	*x*	*y*	*z*	*U*_iso_*/*U*_eq_	
S1	0.07706 (8)	0.29276 (3)	0.01263 (3)	0.02056 (12)	
O1	0.7096 (2)	0.44974 (7)	0.01110 (8)	0.0187 (3)	
N1	0.9164 (3)	0.52631 (8)	0.11276 (10)	0.0179 (3)	
N2	0.3961 (2)	0.43175 (8)	0.17539 (10)	0.0150 (3)	
N3	0.3233 (2)	0.39394 (8)	0.09997 (10)	0.0162 (3)	
N4	0.0280 (2)	0.34699 (8)	0.18614 (10)	0.0159 (3)	
C1	0.6987 (3)	0.51350 (9)	0.24304 (12)	0.0165 (3)	
C2	0.6453 (3)	0.52430 (10)	0.33500 (12)	0.0184 (3)	
H2A	0.5077	0.5027	0.3598	0.022*	
C3	0.7961 (3)	0.56718 (10)	0.39045 (12)	0.0193 (3)	
C4	0.9965 (3)	0.59863 (10)	0.35161 (13)	0.0206 (4)	
H4A	1.0989	0.6276	0.3895	0.025*	
C5	1.0518 (3)	0.58900 (10)	0.25912 (13)	0.0208 (4)	
H5A	1.1879	0.6110	0.2338	0.025*	
C6	0.8998 (3)	0.54610 (10)	0.20639 (12)	0.0169 (3)	
C7	0.7392 (3)	0.47997 (9)	0.08672 (12)	0.0161 (3)	
C8	0.5860 (3)	0.47119 (9)	0.16929 (11)	0.0155 (3)	
C9	0.1363 (3)	0.34475 (9)	0.10484 (11)	0.0149 (3)	
C10	−0.1620 (3)	0.30520 (9)	0.22098 (11)	0.0153 (3)	
C11	−0.3260 (3)	0.26716 (9)	0.16670 (12)	0.0173 (3)	
H11A	−0.3118	0.2663	0.1018	0.021*	
C12	−0.5113 (3)	0.23029 (10)	0.20818 (13)	0.0200 (3)	
H12A	−0.6224	0.2039	0.1711	0.024*	
C13	−0.5366 (3)	0.23132 (10)	0.30262 (13)	0.0230 (4)	
H13A	−0.6641	0.2060	0.3301	0.028*	
C14	−0.3733 (3)	0.26977 (12)	0.35662 (13)	0.0250 (4)	
H14A	−0.3896	0.2711	0.4214	0.030*	
C15	−0.1861 (3)	0.30627 (11)	0.31617 (12)	0.0218 (4)	
H15A	−0.0740	0.3321	0.3535	0.026*	
C16	0.7427 (4)	0.58122 (12)	0.48997 (13)	0.0275 (4)	
H16A	0.5743	0.5738	0.4998	0.041*	
H16B	0.7858	0.6325	0.5061	0.041*	
H16C	0.8332	0.5465	0.5287	0.041*	
H1N4	0.095 (4)	0.3741 (12)	0.2252 (15)	0.021 (5)*	
H1N1	1.034 (4)	0.5376 (13)	0.0759 (16)	0.033 (6)*	
H1N3	0.410 (4)	0.3965 (12)	0.0486 (15)	0.026 (6)*	

### Atomic displacement parameters (Å^2^)

**Table:**

	*U*^11^	*U*^22^	*U*^33^	*U*^12^	*U*^13^	*U*^23^
S1	0.0242 (2)	0.0211 (2)	0.0166 (2)	−0.00282 (16)	0.00367 (15)	−0.00340 (17)
O1	0.0170 (6)	0.0209 (6)	0.0183 (6)	0.0005 (5)	0.0041 (4)	0.0018 (5)
N1	0.0150 (6)	0.0184 (7)	0.0205 (7)	−0.0013 (5)	0.0072 (5)	0.0019 (6)
N2	0.0152 (6)	0.0132 (7)	0.0166 (7)	0.0008 (5)	0.0025 (5)	0.0017 (5)
N3	0.0167 (6)	0.0164 (7)	0.0156 (7)	−0.0017 (5)	0.0048 (5)	−0.0001 (5)
N4	0.0166 (6)	0.0164 (7)	0.0146 (7)	−0.0041 (5)	0.0029 (5)	−0.0003 (6)
C1	0.0147 (7)	0.0148 (8)	0.0199 (8)	−0.0004 (6)	0.0020 (6)	0.0027 (6)
C2	0.0191 (8)	0.0172 (8)	0.0191 (8)	−0.0015 (6)	0.0044 (6)	0.0026 (7)
C3	0.0223 (8)	0.0152 (8)	0.0206 (8)	−0.0003 (6)	0.0015 (6)	0.0008 (7)
C4	0.0206 (8)	0.0162 (8)	0.0250 (9)	−0.0029 (6)	−0.0011 (7)	0.0012 (7)
C5	0.0161 (7)	0.0178 (9)	0.0287 (9)	−0.0025 (6)	0.0042 (6)	0.0014 (7)
C6	0.0148 (7)	0.0148 (8)	0.0212 (8)	0.0015 (6)	0.0040 (6)	0.0026 (6)
C7	0.0148 (7)	0.0135 (8)	0.0204 (8)	0.0028 (5)	0.0041 (6)	0.0055 (6)
C8	0.0136 (7)	0.0156 (8)	0.0174 (8)	0.0007 (6)	0.0038 (6)	0.0038 (6)
C9	0.0154 (7)	0.0137 (8)	0.0157 (8)	0.0001 (6)	0.0014 (6)	0.0025 (6)
C10	0.0137 (7)	0.0137 (8)	0.0186 (8)	−0.0008 (5)	0.0031 (6)	0.0028 (6)
C11	0.0172 (7)	0.0160 (8)	0.0187 (8)	0.0011 (6)	0.0007 (6)	0.0001 (6)
C12	0.0158 (7)	0.0162 (8)	0.0279 (9)	−0.0010 (6)	0.0002 (6)	−0.0001 (7)
C13	0.0166 (8)	0.0206 (9)	0.0319 (10)	−0.0006 (6)	0.0053 (7)	0.0069 (7)
C14	0.0205 (8)	0.0364 (11)	0.0184 (8)	−0.0023 (7)	0.0046 (7)	0.0057 (8)
C15	0.0182 (8)	0.0296 (10)	0.0175 (8)	−0.0038 (7)	0.0011 (6)	0.0013 (7)
C16	0.0351 (10)	0.0269 (10)	0.0205 (9)	−0.0088 (8)	0.0030 (7)	−0.0025 (8)

### Geometric parameters (Å, °)

**Table:**

S1—C9	1.6643 (17)	C4—C5	1.400 (3)
O1—C7	1.236 (2)	C4—H4A	0.9500
N1—C7	1.355 (2)	C5—C6	1.380 (2)
N1—C6	1.414 (2)	C5—H5A	0.9500
N1—H1N1	0.89 (3)	C7—C8	1.507 (2)
N2—C8	1.296 (2)	C10—C11	1.390 (2)
N2—N3	1.349 (2)	C10—C15	1.396 (2)
N3—C9	1.385 (2)	C11—C12	1.392 (2)
N3—H1N3	0.91 (2)	C11—H11A	0.9500
N4—C9	1.346 (2)	C12—C13	1.386 (3)
N4—C10	1.417 (2)	C12—H12A	0.9500
N4—H1N4	0.84 (2)	C13—C14	1.389 (3)
C1—C2	1.393 (2)	C13—H13A	0.9500
C1—C6	1.400 (2)	C14—C15	1.391 (2)
C1—C8	1.454 (2)	C14—H14A	0.9500
C2—C3	1.397 (2)	C15—H15A	0.9500
C2—H2A	0.9500	C16—H16A	0.9800
C3—C4	1.401 (2)	C16—H16B	0.9800
C3—C16	1.508 (3)	C16—H16C	0.9800
			
C7—N1—C6	111.17 (14)	N2—C8—C1	126.19 (15)
C7—N1—H1N1	122.3 (15)	N2—C8—C7	127.40 (16)
C6—N1—H1N1	126.2 (15)	C1—C8—C7	106.37 (13)
C8—N2—N3	117.49 (14)	N4—C9—N3	113.02 (15)
N2—N3—C9	120.16 (14)	N4—C9—S1	129.63 (13)
N2—N3—H1N3	119.0 (14)	N3—C9—S1	117.35 (12)
C9—N3—H1N3	120.3 (14)	C11—C10—C15	119.59 (15)
C9—N4—C10	131.63 (15)	C11—C10—N4	124.33 (15)
C9—N4—H1N4	113.8 (15)	C15—C10—N4	116.02 (15)
C10—N4—H1N4	114.0 (15)	C10—C11—C12	119.45 (16)
C2—C1—C6	120.24 (16)	C10—C11—H11A	120.3
C2—C1—C8	133.01 (15)	C12—C11—H11A	120.3
C6—C1—C8	106.74 (15)	C13—C12—C11	121.24 (17)
C1—C2—C3	119.32 (16)	C13—C12—H12A	119.4
C1—C2—H2A	120.3	C11—C12—H12A	119.4
C3—C2—H2A	120.3	C12—C13—C14	119.17 (17)
C2—C3—C4	118.91 (17)	C12—C13—H13A	120.4
C2—C3—C16	120.98 (16)	C14—C13—H13A	120.4
C4—C3—C16	120.09 (16)	C13—C14—C15	120.20 (17)
C5—C4—C3	122.58 (17)	C13—C14—H14A	119.9
C5—C4—H4A	118.7	C15—C14—H14A	119.9
C3—C4—H4A	118.7	C14—C15—C10	120.33 (17)
C6—C5—C4	117.04 (16)	C14—C15—H15A	119.8
C6—C5—H5A	121.5	C10—C15—H15A	119.8
C4—C5—H5A	121.5	C3—C16—H16A	109.5
C5—C6—C1	121.91 (16)	C3—C16—H16B	109.5
C5—C6—N1	128.63 (15)	H16A—C16—H16B	109.5
C1—C6—N1	109.45 (15)	C3—C16—H16C	109.5
O1—C7—N1	127.33 (15)	H16A—C16—H16C	109.5
O1—C7—C8	126.46 (15)	H16B—C16—H16C	109.5
N1—C7—C8	106.20 (15)		
			
C8—N2—N3—C9	173.20 (15)	C6—C1—C8—N2	178.90 (16)
C6—C1—C2—C3	−0.8 (3)	C2—C1—C8—C7	−177.55 (18)
C8—C1—C2—C3	177.80 (17)	C6—C1—C8—C7	1.20 (18)
C1—C2—C3—C4	0.5 (3)	O1—C7—C8—N2	−1.1 (3)
C1—C2—C3—C16	179.03 (17)	N1—C7—C8—N2	179.90 (16)
C2—C3—C4—C5	0.2 (3)	O1—C7—C8—C1	176.55 (16)
C16—C3—C4—C5	−178.40 (18)	N1—C7—C8—C1	−2.43 (18)
C3—C4—C5—C6	−0.5 (3)	C10—N4—C9—N3	−177.56 (16)
C4—C5—C6—C1	0.1 (3)	C10—N4—C9—S1	2.6 (3)
C4—C5—C6—N1	−178.48 (17)	N2—N3—C9—N4	7.3 (2)
C2—C1—C6—C5	0.5 (3)	N2—N3—C9—S1	−172.89 (12)
C8—C1—C6—C5	−178.41 (16)	C9—N4—C10—C11	−21.0 (3)
C2—C1—C6—N1	179.35 (15)	C9—N4—C10—C15	161.84 (18)
C8—C1—C6—N1	0.42 (19)	C15—C10—C11—C12	−0.4 (3)
C7—N1—C6—C5	176.62 (17)	N4—C10—C11—C12	−177.43 (16)
C7—N1—C6—C1	−2.1 (2)	C10—C11—C12—C13	0.6 (3)
C6—N1—C7—O1	−176.20 (16)	C11—C12—C13—C14	−0.2 (3)
C6—N1—C7—C8	2.77 (18)	C12—C13—C14—C15	−0.4 (3)
N3—N2—C8—C1	−177.47 (15)	C13—C14—C15—C10	0.6 (3)
N3—N2—C8—C7	−0.2 (2)	C11—C10—C15—C14	−0.2 (3)
C2—C1—C8—N2	0.2 (3)	N4—C10—C15—C14	177.06 (17)

### Hydrogen-bond geometry (Å, °)

**Table:**

*D*—H···*A*	*D*—H	H···*A*	*D*···*A*	*D*—H···*A*
N4—H1N4···N2	0.84 (2)	2.14 (2)	2.5947 (18)	114.1 (18)
N1—H1N1···O1^i^	0.89 (2)	1.96 (2)	2.848 (2)	173 (2)
N3—H1N3···O1	0.91 (2)	2.04 (2)	2.7595 (17)	135.8 (19)
C11—H11A···S1	0.95	2.63	3.2712 (18)	125

Symmetry codes: (i) −*x*+2, −*y*+1, −*z*.
